# Oncometabolites and Hypoxia-Regulated Exosomes Shape HIF-Driven Macrophage Programs Across Type 2 Diabetes, Atherosclerosis, and Cancer

**DOI:** 10.3390/ijms27052291

**Published:** 2026-02-28

**Authors:** Antonina Nowinka, Gabriela Krystek, Zuzanna Gontarek, Martyna Góralczyk, Antonina Waligórska, Marta Walenciak, Dorota Formanowicz

**Affiliations:** 1 Faculty of Medicine, Poznan University of Medical Sciences, 60-812 Poznań, Poland; ananowinka@gmail.com (A.N.); gabrielakrystek3@gmail.com (G.K.); zuzanna.gontarek03@gmail.com (Z.G.); goralczykmartyna48@gmail.com (M.G.); antoninawaligorska@gmail.com (A.W.); walenciakmarta123@gmail.com (M.W.); 2 Chair and Department of Medical Chemistry and Laboratory Medicine, Poznan University of Medical Sciences, Rokietnicka 8, 61-701 Poznań, Poland

**Keywords:** hypoxia, HIF-1α, HIF-2α, macrophage polarization, oncometabolites, lactate, succinate, hypoxia-regulated exosomes, type 2 diabetes, obesity, atherosclerosis, tumor microenvironment, TAMs, PI3Kγ, PD-L1, belzutifan

## Abstract

Oncometabolites and hypoxia-regulated exosomes orchestrate hypoxia-inducible factor (HIF)–driven macrophage reprogramming across chronic cardiometabolic and oncologic conditions. In type 2 diabetes (T2D) and obesity, regional hypoxia in expanding white adipose tissue (WAT) reconfigures macrophage immunometabolism and chemokine signaling, recruits C-C chemokine receptor 2 (CCR2^+^) monocytes, and skews adipose-tissue macrophages toward M1-like programs that sustain low-grade inflammation and blunt the physiological M1-to-M2 transition during wound repair. In atherosclerotic plaques, lipid-core hypoxia stabilizes HIF-1α, amplifies nuclear factor kappa-light-chain-enhancer of activated B cells/reactive oxygen species (NF-κB/ROS) signaling, increases matrix metalloproteinase-2/-9 (MMP-2/-9) release, and reduces ATP-binding cassette transporter A1 (ABCA1)-mediated cholesterol efflux, weakening the fibrous cap. In tumors, poorly perfused niches accumulate lactate and succinate, which act as paracrine cues. Lactate activates PKA/cAMP pathways and promotes immunosuppressive tumor-associated macrophages (TAMs), whereas succinate signals through succinate receptor 1 (SUCNR1) to reinforce HIF-1α–dependent transcription and M2-like programming. In parallel, hypoxia-regulated exosomes deliver microRNAs such as miR-301a-3p, which suppress phosphatase and tensin homolog (PTEN) and activate PI3Kγ, thereby augmenting immunosuppression and programmed death-ligand 1 (PD-L1) expression. Clinically, this hypoxia–oncometabolite–exosome triad links oxygen debt with macrophage state, plaque destabilization, impaired wound repair, and tumor immune escape. Translational entry points include selective HIF-2α inhibition, phosphoinositide 3-kinase gamma (PI3Kγ) blockade, SUCNR1 targeting, and exosome-based miRNA modulation, while a biomarker panel comprising HIF-1α, vascular endothelial growth factor A (VEGF-A), and MMP-9 offers a pragmatic readout of hypoxia burden, macrophage programming, and therapeutic response. We conducted a focused narrative review (PubMed, Scopus, Web of Science; English; 2003–2025), prioritizing mechanistic and translational studies on hypoxia–HIF, lactate/succinate, and hypoxia-regulated exosomes across T2D, atherosclerosis, and cancer.

## 1. Introduction

Hypoxia (insufficient tissue oxygenation) reprograms macrophage immunometabolism in type 2 diabetes (T2D), atherosclerosis, and cancer. Through hypoxia-inducible factors (HIF-1α/HIF-2α), cells translate oxygen scarcity into distinct transcriptional programs; HIF-1α predominates in acute hypoxia and drives glycolysis and inflammatory effectors, whereas HIF-2α sustains angiogenic and adaptive outputs under chronic hypoxia—the HIF isoform switch [1,2,3]. These isoform-specific programs reconfigure macrophage states along a continuum that extends beyond the M1/M2 dichotomy, thereby influencing tissue remodeling, inflammation, and immunity [4,5].

Beyond oxygen tension, two hypoxia-linked signals act as potent cross-disease macrophage cues. Lactate accumulates in poorly perfused niches, polarizes tumor-associated macrophages (TAMs), and induces vascular endothelial growth factor-A (VEGF-A) in a HIF-1α-dependent manner; succinate released by cancer cells activates succinate receptor-1 (SUCNR1/G-protein–coupled receptor 91 (GPR91)) and converges on the phosphoinositide 3-kinase (PI3K)–HIF-1α axis to reinforce M2-like programs and migration [6,7,8,9]. In parallel, hypoxia-regulated exosomes deliver microRNA cargo—exemplar microRNA-301a-3p (miR-301a-3p)—that suppresses phosphatase and tensin homolog (PTEN) and activates PI3K-γ (PI3Kγ), augmenting immunosuppression and programmed death-ligand-1 (PD-L1) expression on macrophages [10,11].

Across three high-burden settings, the same template recurs: (1) in expanding white adipose tissue (WAT), regional hypoxia recruits C-C chemokine receptor 2 (CCR2^+^) monocytes and skews adipose-tissue macrophages toward M1-like programs that sustain low-grade inflammation and blunt the physiological M1-to-M2 transition during wound repair [4,12]; (2) in atherosclerotic plaques, lipid-core hypoxia stabilizes HIF-1α, augments glycolysis and matrix metalloproteinase-2/-9 (MMP-2/-9) release, and concurrently reduces ATP-binding cassette transporter A1 (ABCA1)-mediated cholesterol efflux, weakening the fibrous cap [13]; and (3) in tumors, poorly vascularized niches accumulate lactate, succinate, and exosomal microRNAs that polarize tumor-associated macrophages (TAMs) toward immunoregulatory states [6,7,8,9,10,11].

Translational entry points highlighted in this review include selective HIF-2α inhibition (e.g., belzutifan) [14], PI3Kγ blockade [15], targeting SUCNR1 signaling [7], and exosome-based microRNA modulation [11]. As a practical example, we reference a biomarker panel—including HIF-1α, VEGF-A, and MMP-9—that links hypoxia burden with macrophage phenotype and therapeutic response [16]. This systems-level perspective—treating oxidative stress, inflammation, and extracellular matrix (ECM) remodeling as convergent hubs—aligns with the hypoxia–HIF framework and supports operational use of compact biomarker panels in chronic disorders [17,18].

## 2. Search Strategy and Selection Criteria

We conducted a focused, narrative literature review to integrate hypoxia–HIF signaling with lactate/succinate oncometabolites and hypoxia-regulated exosomes as cross-disease macrophage drivers in T2D/obesity, atherosclerosis, and cancer. Sources included PubMed/MEDLINE, Scopus, Web of Science, and Google Scholar. For translational levers, we also screened ClinicalTrials.gov and major oncology meeting abstracts, but only when a corresponding peer-reviewed article already existed. The date window spanned January 2003 to December 2025, with the last comprehensive update on December 28, 2025; language was restricted to English. We targeted mechanistic and translational evidence, including primary experimental studies (in vitro/in vivo), clinical studies (observational and interventional), and high-quality reviews. We excluded case reports without mechanistic content, conference abstracts without accompanying full articles, and non-peer-reviewed preprints unless the findings were subsequently published in a peer-reviewed journal.

Searches combined free text, synonyms, truncations, and MeSH/EMTREE where available, using Boolean logic to ensure sensitivity and specificity. Core constructs were hypoxia, HIF-1α/HIF-2α, macrophage polarization, and immunometabolism (glycolysis vs FAO/OXPHOS), oncometabolites lactate and succinate/SUCNR1, exosomes and miRNA cargo (e.g., miR-301a-3p), PD-L1, and translational levers (HIF-2α inhibitors such as belzutifan, PI3Kγ inhibitors such as eganelisib, SUCNR1 antagonism, exosome-based modulation), plus biomarkers (HIF-1α, VEGF-A, MMP-9) and hypoxia imaging (e.g., [^18F]FMISO PET). Illustrative search strings (adapted to database syntax) were constructed as follows: (“hypoxia” OR “HIF-1α” OR “HIF-2α”) AND (“macrophage*”) AND (“polarization” OR “immunometabolism” OR “glycolysis” OR “FAO” OR “OXPHOS”); (“lactate” OR “succinate” OR “SUCNR1”) AND (“tumor-associated macrophage” OR “TAM” OR “atherosclerosis” OR “wound”); (“exosome” AND “miRNA” AND (“macrophage” OR “TAM”) AND (“PTEN” OR “PI3Kγ” OR “PD-L1”)); (“belzutifan” OR “HIF-2α inhibitor”) AND (“macrophage” OR “TAM”); ([^18F]FMISO OR “hypoxia imaging”) AND (“plaque” OR “tumor”).

Records were de-duplicated and screened at the title/abstract level for relevance to the hypoxia–oncometabolite–exosome triad and macrophage outcomes; full texts were assessed for data richness (pathway mapping, macrophage phenotype readouts, biomarker values, therapeutic lever details). We prioritized studies that reported clear macrophage functional outputs (e.g., M1/M2 continuum features, efferocytosis, cytokine programs, matrix remodeling) and that connected these outputs to hypoxia or metabolite/exosome signals. For translational content, we required explicit mechanistic alignment to myeloid targets (HIF-2α, PI3Kγ, SUCNR1) or exosomal miRNA axes. Data extracted included disease context and stage, tissue oxygenation cues, dominant macrophage programs and metabolic wiring, relevant biomarkers (HIF-1α, VEGF-A, MMP-9 with any reported thresholds), and therapeutic/interventional details with outcome signals; where possible, we noted imaging correlates (e.g., FMISO PET) and assay modalities to inform clinical applicability.

This review is narrative rather than systematic; we did not register a protocol and did not perform a quantitative meta-analysis. Selection bias is possible, particularly toward well-characterized axes in oncology and atherosclerosis; heterogeneity of models and assays limits formal pooling. To mitigate this, we triangulated across multiple databases, favored mechanistically explicit studies and convergent findings across diseases, and anchored recommendations to biomarkers and imaging readouts that can be operationalized in clinical settings.

## 3. Study Aim

To integrate the hypoxia–HIF signaling axis with lactate/succinate oncometabolites and hypoxia-regulated exosomal microRNAs (e.g., miR-301a-3p) as a unifying macrophage-centered framework across T2D, atherosclerosis, and cancer [1,4,5,6,7,10], and to identify translational entry points (HIF-2α inhibition, PI3Kγ blockade, SUCNR1 targeting, exosome-based modulation) [7,8,10,11,14,15] together with a parsimonious biomarker readout (HIF-1α, vascular endothelial growth factor A (VEGF-A), MMP-9) for risk stratification and response monitoring [16].

## 4. Hypoxia–HIF Axis

**Figure 1:**
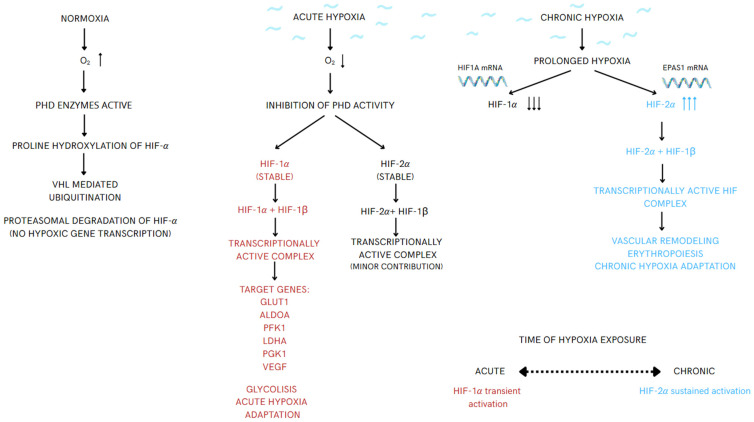
**Temporal regulation of HIF-1α and HIF-2α signaling under hypoxic conditions.** Under normoxic conditions, prolyl hydroxylase domain (PHD) enzymes promote hydroxylation and proteasomal degradation of HIF-α subunits. Acute hypoxia inhibits PHD activity, leading to rapid stabilization of HIF-1α and the formation of transcriptionally active HIF complexes that drive metabolic and angiogenic responses. During prolonged hypoxia, HIF-1α levels decline, whereas HIF-2α remains stabilized and predominates in regulating transcriptional programs associated with chronic hypoxia adaptation. This temporal shift illustrates the HIF isoform switch in response to varying durations of hypoxia. Arrows indicate the direction of regulatory events within the hypoxia response pathway: downward arrows denote reduced oxy-gen availability or inhibitory steps, upward arrows reflect increased expression or activation, and horizontal arrows represent the temporal progression from acute to chronic hypoxia.

Hypoxia is insufficient tissue oxygen that stabilizes hypoxia-inducible factors (HIFs). In normoxia, prolyl hydroxylase domain (PHD) enzymes hydroxylate HIF-α, enabling von Hippel–Lindau (VHL)-mediated proteasomal degradation; under hypoxia, HIF-α escapes degradation, dimerizes with hypoxia-inducible factor 1 beta (HIF-1β) (aryl hydrocarbon receptor nuclear translocator (ARNT)), binds hypoxia-response elements (HREs), and reprograms metabolism and inflammation [1,19]. HIF-1α dominates early/acute hypoxia and drives glycolysis and inflammatory effectors (e.g., inducible nitric oxide synthase (iNOS)/nitric oxide (NO)), whereas HIF-2α sustains chronic adaptations (angiogenesis, survival)—the temporal HIF isoform switch [1,2,3]. In macrophages, this switch enables a continuum of states beyond M1/M2 by tilting metabolism toward glycolysis (M1-like) versus fatty acid oxidation (FAO)/oxidative phosphorylation (OXPHOS) (M2-like) [4,5]. In poorly perfused niches, hypoxia couples with transversal cues—oncometabolites lactate and succinate (via SUCNR1) and hypoxia-regulated exosomes (e.g., miR-301a-3p acting through PI3Kγ)—to amplify HIF programs and shape outcomes across T2D/obesity, atherosclerosis, and cancer (see Figure 1) [6,7,8,9,10,11].

## 5. Transversal Cues Beyond Oxygen: Oncometabolites and Hypoxic Exosomes

Poor perfusion frequently coincides with the accumulation of lactate and succinate, as well as the release of hypoxia-regulated exosomes, forming an oxygen-independent triad that amplifies HIF-driven macrophage programming across T2D/obesity, atherosclerosis, and cancer [6,7,10]. Lactate stabilizes HIF-1α, engages PKA/CREB signaling, and induces VEGF-A and PD-L1 on macrophages, thereby coupling metabolic acidosis with angiogenesis and immune checkpoint up-regulation [6,20]. Succinate acts intracellularly by inhibiting PHDs to stabilize HIF-1α and extracellularly via SUCNR1, where G-protein (Gq) signaling reinforces M2-like transcriptional programs, chemotaxis, and motility [7,8,9]. In parallel, hypoxic exosomes enrich the miRNA cargo—exemplified by miR-301a-3p—which suppresses PTEN and activates PI3Kγ, thereby consolidating immunosuppression and TAM-like polarization [10,11]. These oxygen-independent amplifiers recur across T2D/obesity (hypoxic white adipose tissue, or WAT), atherosclerosis (lipid-core hypoxia), and cancer (poorly perfused niches), providing a unifying layer that magnifies the HIF-driven reprogramming outlined in the Introduction.

## 6. Type 2 Diabetes and Obesity: The Hypoxia–HIF–Macrophage Axis

### 6.1. Global Burden of Type 2 Diabetes (2024–2050)—Clinical Rationale for the Hypoxia–HIF–Macrophage Axis

In 2024, ~589 million adults (20–79 years) had diabetes, ~81% of cases occurred in low- and middle-income countries, and ~3.4 million deaths were attributed to diabetes in 2024; global diabetes-related health expenditure exceeded USD 1.0 trillion (~12% of health spending) [21,22,23]. These magnitudes—especially underdiagnosis and the LMIC concentration—justify a mechanistic focus on oxygen debt and macrophage programming: regional hypoxia in expanding adipose tissue and in chronic wounds stabilizes HIF-1α/HIF-2α [1,2,3,24,25,26,27] and, together with lactate [6], succinate/SUCNR1 [7,8] and hypoxia-regulated exosomes [10,11], locks inflammatory macrophage states that worsen insulin resistance, impair wound repair and amplify downstream complications; see Table 1 for a concise cross-condition map of hypoxia-induced pathways, immunometabolism, and macrophage polarization.

In the following Section 6.2, Section 6.3, Section 6.4, Section 6.5, Section 6.6, Section 6.7 and Section 6.8 [26,27], we map how adipose and wound hypoxia translate into macrophage immunometabolism and clinical trajectories in T2D.

### 6.2. Adipose Tissue Hypoxia and the HIF Switch in T2D

Excessive expansion of white adipose tissue (WAT) creates a mismatch between tissue growth and vascularization, leading to regional hypoxia. This oxygen deficit initiates a temporal switch in HIF isoforms, with HIF-1α dominating during early, acute hypoxia and HIF-2α supporting chronic adaptive responses [1,2,3,24]. Under these conditions, hypoxia reprograms adipocyte metabolism and alters adipokine secretion, raising leptin, IL-6, and TNF-α while reducing adiponectin, and directly impairs insulin signaling in fat cells. These changes collectively contribute to systemic metabolic dysfunction and the development of T2D [24,25].

Within this hypoxic niche, CCR2^+^ monocyte recruitment and the expansion of adipose-tissue macrophages (ATMs) sustain low-grade inflammation along a continuum that extends beyond the M1/M2 stereotype; HIF–1α–driven immunometabolic wiring tilts macrophages toward glycolysis-biased inflammatory states [4,12,28,29,30]. The same hypoxia-centered logic links adipose inflammation to impaired wound-repair trajectories in diabetes, in which persistent HIF-1α activity contributes to a failure of the M1-to-M2 transition [26,27].

#### 6.2.1. Hypoxia in Obesity

Obesity is a major risk factor for T2D and related complications. The expansion of white adipose tissue (WAT) creates a poorly perfused, low-oxygen microenvironment that promotes adipocyte dysfunction and chronic, low-grade inflammation [24]. Vascular adaptation is insufficient compared with adipocyte hypertrophy, resulting in persistent regional hypoxia, which, in turn, feeds forward into dysregulated adipokine secretion and insulin resistance [24]. Human data confirm adipokine–lipid/glucose coupling in obesity (chemerin–triglycerides (TG), OGTT 120′; adipolin–LDL-C), reinforcing WAT dysfunction as a systemic driver [28].

#### 6.2.2. Influence of Hypoxia on Macrophages in Adipose Tissue

The proportion of adipose-tissue macrophages (ATMs) increases markedly in obesity, rising from less than 10% of stromal vascular cells in lean adipose tissue to approximately 50% in obese depots [29]. Chemotactic cues released by preadipocytes and adipocytes—including MIF—and by hypoxic WAT (MCP-1/CCL2, MIP-1α/CCL3) drive the recruitment of circulating monocytes [30,31]. Recruited CCR2^+^ monocytes preferentially differentiate toward pro-inflammatory M1-like macrophages, shifting the balance away from M2-like states observed in lean adipose tissue; genetic disruption of CCR2 limits this influx and preserves a more reparative phenotype [12]. Importantly, single-phenotype labels are simplistic—ATMs exist along a phenotypic continuum—but hypoxia consistently biases these cells toward inflammatory programs [31]. Functionally, increased secretion of TNF-α and other cytokines by ATMs exacerbates insulin resistance at both the tissue and systemic levels [29].

### 6.3. Oncometabolites in Diabetic Adipose Tissue: Succinate and Lactate as Paracrine Macrophage Cues

Succinate accumulates in hypoxic or hyperglycemic adipose tissue and signals via SUCNR1 on macrophages to modulate chemotaxis and polarization, with context-dependent outputs reflecting state-specific receptor wiring [32,33,34]. Lactate acts as a bona fide paracrine metabolite: perturbed lactate handling in adipocytes can trigger apoptosis and cytokine release, recruit macrophages, and thereby promote systemic insulin resistance, mirroring the same HIF-centered template observed in atherosclerosis and cancer [35,36,37].

### 6.4. Pancreatic Islets: ARNT/HIF-1β, HIF-1α, and First-Phase GSIS

In people with T2D, ARNT/HIF-1β mRNA is reduced in pancreatic islets, and experimental HIF-1α deficiency in β-cells blunts the first-phase GSIS by limiting glycolytic ATP and impairing stimulus–secretion coupling—the ~10 min early insulin burst that stabilizes post-prandial glycemia [38,39].

Low HIF-1α activity reduces glycolytic flux and aggravates glycemic control [40]. Hypoxia–inflammation crosstalk within the islet milieu contributes to the development of chronic complications [41,42]. In adipose tissue, hypoxia alters adipokine profiles—suppressing insulin-sensitizing signals while upregulating pro-inflammatory mediators—and attenuates insulin-receptor signaling, thereby undermining systemic glycemic control [43,44]. Within islets and adipose depots, obesity-associated inflammation recruits circulating monocytes, expands crown-like structures, and biases macrophages toward an M1-like phenotype [45,46]. Endocrine dysfunction is amplified by impaired first-phase GSIS and intermittent-hypoxia/HIF-1–dependent NADPH oxidase 4 (NOX4)/H_2_O_2_ signaling, which blunts stimulus–secretion coupling and worsens post-prandial glycemia [47,48]. As insulin sensitivity deteriorates, IL-1β and TNF-α levels rise, thereby consolidating the inflammatory loop across the islet and adipose microenvironments [49].

HIF-1α is the central regulator of these changes: in adipocytes, it drives hypoxia-dependent insulin resistance, while in β-cells it integrates oxygen shortage with the remodeling of glycolysis, ATP generation, and redox-dependent stimulus–secretion coupling [40,43,48]. In parallel, endocrine dysfunction is amplified by impaired first-phase GSIS and by intermittent hypoxia/HIF-1–dependent NOX4/H_2_O_2_ signaling, which blunts stimulus–secretion coupling and worsens postprandial glycemia [47,48]. Together, these islet–adipose interactions consolidate a feed-forward loop of metabolic inflammation that accelerates cardiometabolic and microvascular risk [45,46,47,48,49].

**Figure 2:**
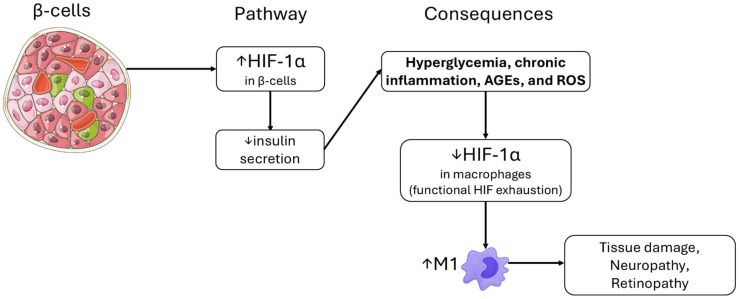
**Pancreatic β-cell hypoxia impairs first-phase GSIS via HIF-1α-driven metabolic reprogramming and contributes to systemic inflammatory complications.** Hypoxia in pancreatic β-cells stabilizes HIF-1α, rewiring glycolysis and diminishing ATP-dependent stimulus–secretion coupling, which leads to impaired first-phase glucose-stimulated insulin secretion (GSIS) and reduced insulin output. The resulting hyperglycemia exacerbates oxidative and inflammatory stress, promoting the accumulation of advanced glycation end products (AGEs), reactive oxygen species (ROS), and pro-inflammatory cytokines. These systemic cues contribute to functional HIF-1α exhaustion in macrophages, lowering HIF-1α activity and skewing macrophages toward a persistent M1-dominant phenotype. M1 polarization drives tissue-damaging inflammatory programs that underlie microvascular diabetic complications, including neuropathy and retinopathy. Parts of the figure were created using images provided by Servier Medical Art (https://smart.servier.com), licensed under CC BY 4. (https://creativecommons.org/licenses/by/4.0/) (Accessed 19 February 2026).

In summary, Figure 2 synthesizes this HIF-1α–centered framework, illustrating how hypoxia in pancreatic β-cells increases HIF-1α levels, impairs first-phase GSIS, and drives hyperglycemia. The resulting metabolic and inflammatory stress—characterized by elevated levels of advanced glycation end products (AGEs), reactive oxygen species (ROS), and cytokine tone—contributes to functional HIF-1α exhaustion in macrophages, thereby promoting sustained M1 polarization. Together, β-cell dysfunction and macrophage-driven inflammation reinforce dysglycemia and accelerate microvascular complications. Clinically, this supports strategies that normalize HIF-1α signaling and β-cell redox coupling (e.g., modulation of HIF-1α/NOX4 pathways), reduce systemic and adipose tissue hypoxia (weight loss, treatment of sleep-disordered breathing), and dampen islet/adipose inflammation (e.g., IL-1β blockade or macrophage-targeted approaches) to improve metabolic control and protect against microvascular disease [40,43,44,47,48,49].

Normoxia: glucose uptake activates glycolysis and OXPHOS, generating an ATP rise that closes ATP-sensitive K^+^ (KATP) channels, triggers Ca^2+^ influx, and drives prompt first-phase insulin release.

Hypoxia/HIF-1α activation: inhibition of pyruvate dehydrogenase (PDH), reduced mitochondrial flux, and ATP shortfall blunt stimulus–secretion coupling and impair first-phase GSIS. The resulting hyperglycemia amplifies oxidative and inflammatory processes (AGEs, ROS), contributing to islet inflammation and increasing the risk of microvascular complications such as neuropathy and retinopathy [38,39,43,48].

### 6.5. Diabetic Wound Healing: Macrophage Phenotype Dysregulation Under Hypoxia/Inflammation

Chronic diabetic wounds exhibit persistent M1-like dominance with failure of the normal M1-to-M2 transition required for resolution and repair; hyperglycemia, AGEs, TLR tone, and regional hypoxia sustain HIF-1α and glycolytic programs, locking wounds in non-healing trajectories [50,51,52]. In contrast to normal closure—where efferocytosis, interleukin-10 (IL-10) and FAO/OXPHOS-driven M2 functions restore tissue integrity—diabetic ulcers show M1-driven cytokine release, matrix damage, delayed epithelialization and poor angiogenesis, consistent with impaired macrophage phenotype switching across later healing phases [53,54,55].

Clinically, correcting the macrophage M1-to-M2 imbalance improves healing outcomes in diabetic foot ulcers. In a multicenter, evaluator-blinded phase 3 trial, topical ON101 significantly increased complete healing compared with standard dressings [56].

In routine healing, transient hypoxia stabilizes HIF-1α, wiring macrophages for early microbicidal functions (glycolysis, ROS/nitric oxide (NO)) and then resolves toward reparative M2-like programs supported by FAO/OXPHOS [4,52]. In turn, in diabetes, persistent hypoxia and inflammatory tone (advanced glycation end-products, AGEs; Toll-like receptors, TLRs) sustain HIF-1α, hinder efferocytosis, and lock wounds in an M1-dominant state that damages ECM and stalls granulation [26,27]. Poor perfusion and hyperglycemia favor the accumulation of lactate and succinate in chronic wounds. Lactate amplifies HIF-1α signaling through the protein kinase A–cAMP response element-binding protein (PKA–CREB) pathway. In contrast, succinate activates SUCNR1 on macrophages, reinforcing chemotaxis and polarization toward non-resolving inflammation [6,7,8,35]. These paracrine loops help explain the persistent M1 dominance and impaired angiogenesis in diabetic ulcers [26,27,52]. Hypoxic tissues increase exosome biogenesis and loading of immunomodulatory miRNAs. In tumors, miR-301a-3p suppresses PTEN and activates PI3Kγ in macrophages, promoting immunosuppression [10,11]. We list this axis as a mechanistic hypothesis for chronic diabetic wounds that share hypoxic and inflammatory features [52]. This framework explains the entries in Table 2 across the phases (inflammation, proliferation, and remodeling) and motivates therapeutic levers that reduce oxygen debt, rebalance immunometabolism, and protect matrix integrity [52,56]. Reducing wound hypoxia further supports macrophage M1-to-M2 switching and granulation: sustained oxygenation devices accelerate epithelialization/angiogenesis and dampen cytokine tone [57]. Mechanistically, the selective inhibition of MMP-9 preserves ECM architecture and facilitates effector T-cell trafficking, offering a tractable adjunct to curb non-resolving inflammation in chronic diabetic ulcers [58].

Moreover, SGLT2 inhibitors improve endothelial function and NO bioavailability, providing microvascular relief [59,60]. In addition, GLP-1 signaling favorably modulates lipid handling (e.g., ABCA1/SR-B1 and cholesterol efflux), which can indirectly rebalance macrophage programs in metabolically inflamed tissues [61]. Beyond glucose-lowering, GLP-1 receptor agonists exert central and peripheral anti-inflammatory actions that dampen TLR-driven responses and improve cardiometabolic risk, providing a mechanistic rationale for their inclusion as de-inflammation levers in chronic wounds [62,63]. In parallel, SGLT2 inhibitors attenuate macrophage-mediated inflammation (inflammasome and TLR4/NF-κB activity; M1-to-M2 rebias), complementing the microvascular benefits and supporting their phase-specific use in diabetic wound care [64].

**Table 2:** Macrophage dynamics in diabetic wound healing: hypoxia–HIF–metabolic reprogramming across phases (normal vs diabetic).

Wound-Healing Phase	Local Oxygen/HIF Status	Dominant Macrophage Phenotype	Key Signals (Examples)	Metabolic Program	Principal Effector Functions	Clinical Outcome	Potential Therapeutic Levers	References
Inflammation (normal)	Transient hypoxia; early HIF-1α activation that subsequently resolves	M1-like (early, microbicidal)	DAMPs/TLR signals, IFN-γ; controlled ROS/NO synthesis	Increased Glycolysis (HIF-1α- dependent)	Pathogen clearance; debris removal	Progression to proliferative phase	Timely debridement; infection control	[4,52]
Inflammation (diabetic)	Persistent hypoxia, AGEs sustained, TLR tone; prolonged HIF-1α activity	M1-dominance (failure to initiate resolution)	AGEs, succinate-SUCNR1 signaling, lactate-PKA–CREB–HIF-1α pathway	Increased glycolysis; decreased efferocytosis	High pro- inflammatory cytokines; ECM damage	Chronic non- healing inflammatory state	Oxygen therapy SGLT2 inhibitors (microvascular, anti-inflammatory action)	[6,7,8,26,27,35,57,59,60,64]
Proliferation (normal)	Re-oxygenation established; balanced HIF-2α and pro-angiogenic signals	M2-like (reparative)	IL-4, IL-13, IL-10; efficient efferocytosis	Increased FAO/OXPHOS	Angiogenesis, collagen deposition, and granulation	Timely wound closure	Supportive wound care; optimization of perfusion	[4,52]
Proliferation (diabetic)	Hypoxia persists; lactate/succinate paracrine loops remain active	M1 > M2 (switch failure)	Upregulation of PD-L1 on TAM-like macrophages; exosomal miR-301a-3p suppressing PTEN and enhancing PI3Kγ signaling	Glycolysis-biased metabolism; insufficient FAO/OXPHOS	Impaired angiogenesis; stalled granulation tissue formation	Delayed healing	GLP-1 RAs (anti- inflammatory and; lipid handling effects) targeting SUCNR1 or PI3Kγ	[10,20,32,61,62,63]
Remodeling (normal)	Normoxia restored	M2-like maintained	Pro- resolving mediators	FAO/OXPHOS	ECM remodeling; buildup of tensile strength	Normal scar maturation	Standard care	[4,52]
Remodeling (diabetic)	Persistent micro-foci of hypoxia	M1 persistence; M2 deficit	Increased MMP-9; elevated ROS; unresolved inflammation	Increased glycolysis; inefficient mitochondrial function	ECM breakdown; tissue fragility	Recurrent ulceration	Anti-MMP-9 strategies; perfusion/oxygenation support	[26,57,58]

*Abbreviations:* AGEs—advanced glycation end-products; Ca^2+^—calcium ion; DAMPs—damage-associated molecular patterns; ECM—extracellular matrix; FAO—fatty-acid oxidation; HIF-1α—hypoxia-inducible factor-1 alpha; HIF-2α—hypoxia-inducible factor-2 alpha; IL-4—interleukin-4; IL-10—interleukin-10; IL-13—interleukin-13; MMP-9—matrix metalloproteinase-9; NO—nitric oxide; OXPHOS—oxidative phosphorylation; PD-L1—programmed death-ligand 1; PKA–CREB—protein kinase A/cAMP response element-binding protein pathway; PTEN—phosphatase and tensin homolog; ROS—reactive oxygen species; SGLT2—sodium–glucose cotransporter-2; SUCNR1—succinate receptor-1; TAM—tumor-associated macrophage; TLR—Toll-like receptor.

Accordingly, Table 2 maps oxygen/HIF cues, macrophage states, and immunometabolic wiring across phases and links them to actionable levers—oxygenation [57], anti-MMP-9 [58], SGLT2 inhibitors [59,60], and GLP-1 receptor agonists (de-inflammation and lipid handling via ABCA1/SR-B1) [61,62,63]—to guide phase-specific intervention.

### 6.6. Intestinal Microbiota–Macrophage Axis in T2D

The intestinal microbiota modulates metabolic and immune pathways that shape macrophage activation and insulin sensitivity in T2D [65,66]. Reduced abundance of beneficial taxa (e.g., *Bifidobacterium*, *Bacteroides*, *Faecalibacterium*, *Roseburia*) and expansion of pathobionts (e.g., *Fusobacteria*, *Ruminococcus*, *Blautia*) favor chronic low-grade inflammation, macrophage M1-skewing, and impaired glycemic control [65,67]. Mechanistically, altered microbial metabolites (e.g., SCFAs vs. endotoxin) intersect with adipose hypoxia/HIF signaling, reinforcing pro-inflammatory macrophage programs and insulin resistance [68,69,70,71,72]; conversely, pre/probiotics and glucose-lowering therapies that improve the microenvironment (e.g., SGLT2 inhibitors) may secondarily de-inflate macrophage tone [64,67,73,74,75].

### 6.7. Therapeutic Implications: Dampening Hypoxia Burden and Reprogramming Macrophage Signals

GLP-1 receptor agonists not only improve glycemia and weight but also exert anti-inflammatory actions and favor cholesterol handling in metabolic tissues (↑ ABCA1/SR-B1) via direct macrophage signaling and a neuro-immune axis, thereby de-inflaming T2D microenvironments [61,62,63].

SGLT2 inhibitors improve endothelial function (e.g., FMD), enhance NO bioavailability, and attenuate ROS/inflammation, mechanisms that reduce microvascular hypoxia and secondarily dampen the HIF-macrophage drive [59,60]. Targeting succinate–SUCNR1 and exosome–PI3Kγ nodes provides tractable entry points to limit metabolite-driven macrophage dysregulation in T2D and to counter myeloid-mediated resistance in hypoxic tumors [10,15,32].

Endothelial support and oxygenation devices reduce hypoxia burden, facilitating the M1-to-M2 transition [57]. SGLT2 inhibitors improve microvascular function and lower inflammatory/oxidative tone [59,60], while GLP-1 receptor agonists exert central and peripheral anti-inflammatory effects and can favor cholesterol handling (ABCA1/SR-B1) [61,62,63], thereby de-inflaming the wound microenvironment. In matrix-fragile ulcers, anti-MMP-9 strategies are a rational adjunct to protect the ECM while optimizing perfusion [58]. These interventions align with the phase-specific entries listed in Table 2.

### 6.8. Synthesis and Biomarker Readout

Across T2D, atherosclerosis, and cancer, hypoxia-linked metabolites—lactate and succinate—and hypoxic exosomes rewire immunometabolism and program macrophages along disease-relevant continua that govern tissue remodeling, inflammation, and immunity [4,76,77]. A pragmatic biomarker triad (HIF-1α, VEGF-A, MMP-9) links hypoxia burden to macrophage state and correlates with plaque vulnerability and tumor aggressiveness [16,58,78]. Hypoxia stabilizes HIF-1α/HIF-2α [79]. The triad also provides an operational bridge to therapy selection and response monitoring, including post-stroke recovery [80]. Systems modeling prioritizes oxidative/PKC modules as upstream drivers, consistent with this biomarker triad [81].

## 7. Atherosclerosis—Hypoxia–HIF–Macrophage Axis

Atherosclerosis is a chronic inflammatory and oxidative stress condition driven by abnormal lipid metabolism and immunologic reactions to self-components in the arterial wall [19]. It underlies ischemic heart disease and stroke, leading to global causes of mortality; risks include LDL cholesterol, hypertension, diabetes, smoking, age, male sex, and family history [19].

### 7.1. Global Burden of Atherosclerosis—Clinical Rationale for Hypoxia–Macrophage Axis

Atherosclerosis underlies ischemic heart disease and stroke, which together account for approximately 19.8 million deaths annually, representing nearly 32% of all global deaths [82]. Cardiovascular disease prevalence reached ~612 million cases worldwide, with ~254 million individuals affected by ischemic heart disease and ~94 million by stroke [83]. Coronary artery disease alone impacts over 200 million people, and peripheral artery disease affects more than 230 million adults globally. Despite advances in lipid-lowering therapy, residual risk remains high, particularly in patients with diabetes, obesity, and chronic kidney disease. These magnitudes justify a mechanistic focus on hypoxia-driven macrophage programming and plaque vulnerability as targets for precision intervention.

Carotid intima–media thickness (IMT) serves as a non-invasive marker of burden; importantly, statin therapy slows IMT progression, with the greatest benefit when initiated during phases of accelerated plaque growth [84].

### 7.2. Pathomechanism of Atherosclerotic Plaque Formation

Subendothelial LDL retention (binding to proteoglycans), oxidative modification to Ox-LDL, and monocyte recruitment initiate a cascade leading to foam cells, cytokine release, further LDL oxidation, SMC proliferation, and collagen deposition [13,85]. ROS amplify cell death and cap fragility; platelet activation exacerbates endothelial dysfunction and NO deficiency, perpetuating disease progression [19].

### 7.3. Macrophage Polarization

**Figure 3:**
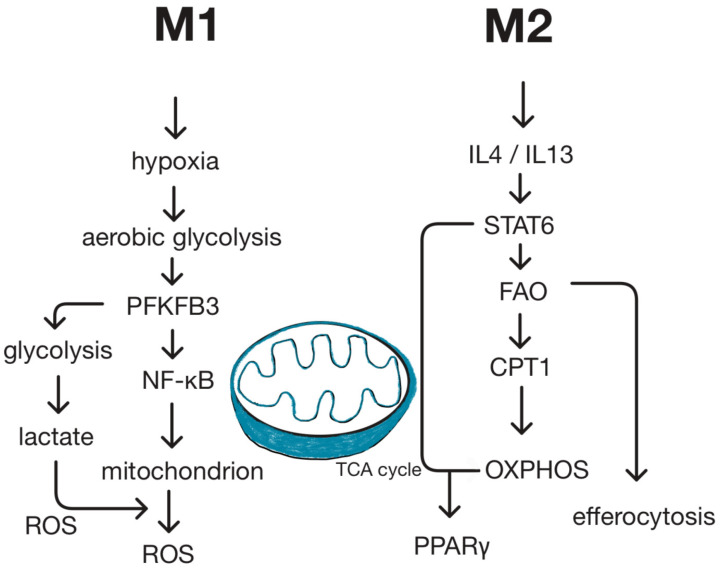
**Divergent Mitochondrial and Metabolic Pathways Underlying M1 and M2 Phenotypes.** M1 macrophages (pro-inflammatory) undergo a metabolic shift toward aerobic glycolysis, driven by hypoxia-inducible factor-1α (HIF-1α) stabilization and upregulation of 6-phosphofructo-2-kinase/fructose-2,6-bisphosphatase 3 (PFKFB3). This process limits mitochondrial tricarboxylic acid (TCA) cycle activity, increases lactate production, and elevates reactive oxygen species (ROS), thereby activating nuclear factor-κB (NF-κB) and promoting pro-inflammatory gene expression. In contrast, M2 macrophages (anti-inflammatory) rely on fatty-acid oxidation (FAO) and oxidative phosphorylation (OXPHOS). Stimulation by interleukin-4/interleukin-13 (IL-4/IL-13) activates the signal transducer and activator of transcription 6 (STAT6) pathway, enhancing carnitine palmitoyltransferase 1 (CPT1)-dependent mitochondrial respiration and peroxisome proliferator-activated receptor-γ (PPARγ) activity. This efficient bioenergetic state supports long-term reparative functions, including tissue remodeling and efferocytosis.

Macrophage activation states are closely linked to their underlying metabolic programs. Pro-inflammatory M1 macrophages adopt a glycolytic phenotype driven by hypoxia-inducible factor-1α (HIF-1α), whereas alternatively activated M2 macrophages rely on fatty-acid oxidation (FAO) and mitochondrial oxidative phosphorylation (OXPHOS) induced by interleukin-4/interleukin-13 (IL-4/IL-13)–STAT6 signaling. These divergent bioenergetic states support the distinct effector functions of M1 and M2 cells. The principal metabolic pathways governing this polarization are summarized in Figure 3.

### 7.4. Impact of Hypoxia on Macrophage Polarization in Plaque

#### 7.4.1. Metabolic Layer: Glycolysis–FAO/OXPHOS Dichotomy in Plaque Macrophages

**Figure 4:**
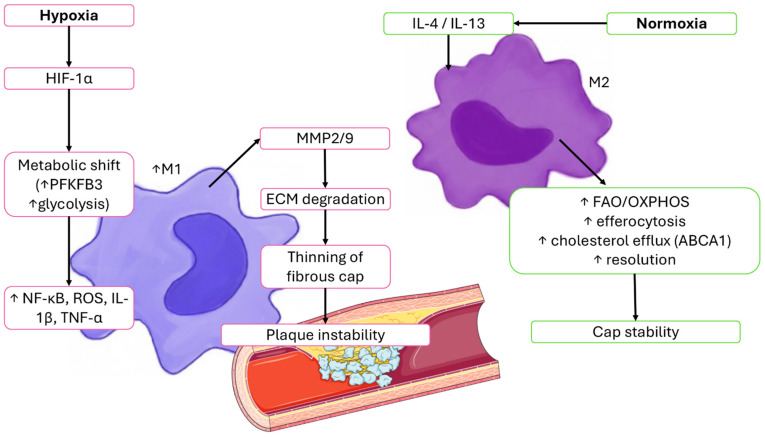
**Hypoxia-driven M1 macrophage programming and normoxia-supported M2 functions in atherosclerosis.** Hypoxia in lipid-rich, poorly perfused regions of the atherosclerotic plaque stabilizes hypoxia-inducible factor-1α (HIF-1α), inducing a glycolytic metabolic shift via 6-phosphofructo-2-kinase/fructose-2,6-bisphosphatase 3 (PFKFB3) and enhancing glycolysis. This promotes polarization toward M1 macrophages, which activate nuclear factor-κB (NF-κB), generate reactive oxygen species (ROS), and produce pro-inflammatory cytokines such as interleukin-1β (IL-1β) and tumor necrosis factor-α (TNF-α). These mediators upregulate matrix metalloproteinases-2 and -9 (MMP-2/9), driving extracellular matrix (ECM) degradation, thinning of the fibrous cap, and plaque instability. In contrast, better-oxygenated plaque regions support M2 macrophages, whose polarization is reinforced by interleukin-4 (IL-4) and interleukin-13 (IL-13). M2 cells rely on fatty-acid oxidation (FAO) and oxidative phosphorylation (OXPHOS), exhibit efficient efferocytosis, and enhance cholesterol efflux through ATP-binding cassette transporter A1 (ABCA1). These pro-resolving functions promote extracellular matrix repair and stabilize the fibrous cap. Parts of the figure were created using images provided by Servier Medical Art (https://smart.servier.com), licensed under CC BY 4. (https://creativecommons.org/licenses/by/4.0/) (Accessed 19 February 2026).

Key mechanisms by which hypoxia shapes macrophage behavior in atherosclerotic lesions are summarized in the subsections below. A hypoxia-driven immunometabolic overview of atherosclerotic plaque macrophages is shown in Figure 4.

Macrophage bias in hypoxic plaque regions may be further reinforced by metabolite–receptor signaling, notably succinate–SUCNR1, paralleling observations in tumors and adipose tissue [34,76].

Hypoxia-driven metabolic rewiring creates a functional dichotomy between plaque macrophages. M1 cells, enriched in glycolysis and NF-κB/ROS signaling, predominate at vulnerable shoulders and secrete MMP-2 and MMP-9, weakening the fibrous cap and promoting instability. Conversely, M2 macrophages rely on FAO/OXPHOS, support efferocytosis and collagen deposition, and localize to better-oxygenated regions, contributing to relative stability. This spatial and metabolic polarization underpins plaque vulnerability and clinical risk, reinforcing the rationale for therapeutic strategies that rebalance macrophage programs [5,13,20].

**Figure 5:**
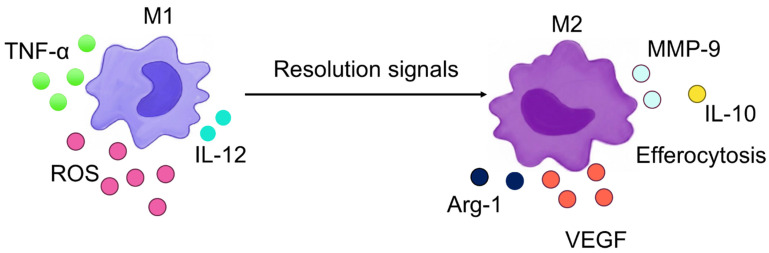
**Classical macrophage polarization from M1 to M2 in response to pro-resolving signals.** The diagram illustrates the characteristic cytokine and effector profiles that define classically activated M1 macrophages and alternatively activated, reparative M2 macrophages. M1 macrophages release pro-inflammatory mediators, including interleukin-12 (IL-12), tumor necrosis factor-α (TNF-α), and reactive oxygen species (ROS), which sustain microbicidal and inflammatory programs. In contrast, M2 macrophages secrete interleukin-10 (IL-10), arginase-1 (Arg-1), vascular endothelial growth factor (VEGF), and matrix metalloproteinase-9 (MMP-9), and they engage in efferocytosis, supporting tissue repair, angiogenic balance, and resolution of inflammation. The transition from M1- to M2-like states is promoted by pro-resolving signals, which rebalance immunometabolism from glycolysis-biased inflammatory activity toward FAO/OXPHOS-supported reparative functions.

The canonical cytokine and effector profiles of M1 and M2 macrophages are summarized in Figure 5, which illustrates the shift from glycolysis-biased, pro-inflammatory M1 programs (IL-12, TNF-α, ROS) toward FAO/OXPHOS-supported reparative M2 states characterized by IL-10, Arg-1, VEGF, MMP-9, and efferocytosis.

#### 7.4.2. Epigenetic Layer: Histone Acetylation Under Hypoxia

Beyond metabolic fluxes, hypoxia and lactate establish an epigenetic layer in macrophages. In hypoxic, lactate-rich niches, histone lactylation emerges as a chromatin signal that reinforces pro-angiogenic and immunoregulatory programs, including PD-L1 upregulation and M2/M2d-like features, as documented in TAMs. By analogy, similar lactate-linked epigenetic cues are poised to operate in hypoxic microdomains of atherosclerotic plaques [20,76]. Functionally, such lactate-epigenetic coupling may intensify matrix remodeling (e.g., MMP-2/-9) and cap weakening in regions with glycolysis-biased M1 signatures, while stabilizing reparative outputs in better-oxygenated regions that sustain FAO/OXPHOS-driven M2 functions [5,13]. This epigenetic add-on complements the HIF-centric immunometabolic model, nominating lactate handling and chromatin modifiers as adjunct translational levers in plaque biology [20,76].

### 7.5. Hypoxia Biomarkers and Imaging (Plaque Vulnerability)

#### 7.5.1. Clinical Correlation and Risk Stratification

An integrated panel (HIF-1α, VEGF-A, MMP-9) links hypoxia burden to macrophage state and clinical risk: MMP-9 elevation predicts obstructive coronary disease, while VEGF associations help refine stratification [16]. A combined analysis of protein/mRNA (MMP-9, VEGF) also correlates with functional recovery in neurovascular settings [80]. Non-invasive hypoxia imaging with [^18F]FMISO PET can localize hypoxic plaque regions and align with immunometabolic activity [86]. Mechanistic and risk-trajectory insights complement modeling of IMT thickening and macrophage dynamics in atherosclerosis [81]. Operationally, integrating this panel with carotid IMT trajectories offers a pragmatic bridge from biology to bedside risk stratification [84,85]. Given these hypoxia-driven macrophage dynamics, pairing [^18F]FMISO PET (to localize hypoxic plaque niches) with the HIF-1α/VEGF-A/MMP-9 panel may personalize treatment intensity and plaque reprogramming monitoring [86].

#### 7.5.2. Imaging-Guided Strategies: FMISO PET and Biomarker Integration

Hypoxia imaging using ^18F-fluoromisonidazole positron emission tomography (FMISO PET) adds a spatial dimension to biomarker-based risk stratification [86]. This technique enables non-invasive localization of hypoxic plaque niches that align with glycolysis-biased M1 macrophage programs and matrix-degrading activity [86]. Although diagnostically valuable, FMISO PET faces practical limitations: the tracer demonstrates slow uptake and clearance, requiring delayed imaging—typically 2 to 4 hours post-injection—to achieve reliable contrast between normoxic and hypoxic tissues. Its availability is further constrained by the need for on-site cyclotron facilities and specialized distribution networks, while high synthesis and operational costs limit routine clinical adoption. Clinically, FMISO PET supports precision mapping of hypoxia in tumors and atherosclerotic plaques, informing radiotherapy planning and identifying vulnerable regions associated with macrophage-driven inflammation and extracellular matrix remodeling [86]. Coupling this imaging modality with the biomarker triad (HIF-1α, VEGF-A, MMP-9) provides a dual readout of oxygen debt and macrophage state, enabling personalized therapy intensity [17]. Conceptually, FMISO PET-positive segments combined with elevated MMP-9 and VEGF-A could nominate candidates for adjunct interventions beyond lipid lowering—such as PI3Kγ or SUCNR1 modulation and PPARγ-driven FAO/OXPHOS support—to stabilize vulnerable plaques [17]. This imaging–biomarker synergy also provides a platform for monitoring macrophage reprogramming and therapeutic response longitudinally [81].

Beyond risk stratification, FMISO PET provides an opportunity to monitor therapeutic response over time. Serial imaging can capture dynamic changes in hypoxic burden following interventions such as HIF pathway inhibitors, PI3Kγ blockade, or metabolic modulators, offering a functional correlate to biomarker trends [86]. This longitudinal approach enables clinicians to assess whether targeted strategies effectively reduce oxygen debt and rebalance macrophage programs, thereby supporting adaptive treatment decisions. Integrating FMISO PET with the biomarker triad strengthens this monitoring framework, creating a combined spatial and molecular readout that aligns with precision medicine principles [17,81].

### 7.6. Translational Targets in Plaque

Translational strategies in plaque biology converge on mechanisms that reduce hypoxia burden and rebalance macrophage immunometabolism. Improving endothelial function and nitric oxide bioavailability enhances perfusion and dampens microhypoxia, thereby mitigating inflammatory drive [46]. Modulation of the HIF-1α pathway represents another critical lever, as it curbs glycolysis-linked M1 programs and limits the matrix-degrading cascade mediated by metalloproteinases [5]. Supporting efferocytosis and oxidative metabolism through PPARγ activation and mitochondrial biogenesis sustains reparative M2 functions, promoting plaque stability [13]. In parallel, the selective inhibition of MMP-9 offers a rational approach to preserve the fibrous cap and restrict immune cell trafficking, thereby reducing vulnerability to rupture [58]. At the population level, statin therapy aligns with logistic models of intima–media thickness progression, reinforcing the importance of early intervention during phases of accelerated plaque growth [84].

#### 7.6.1. Succinate–SUCNR1 Signaling in Atherosclerotic Plaque Macrophages

Hypoxic and necrotic plaque microdomains are poised to accumulate succinate, a metabolite that, in myeloid cells, signals through SUCNR1 (GPR91) to promote chemotaxis and polarization via Gq-coupled pathways. This mechanism is well-documented in cancer and metabolic tissues and is mechanistically compatible with hypoxic plaque macrophages [7,8,9]. Conceptually, SUCNR1 antagonism (or biased modulators) could limit metabolite-reinforced M1/M2 mis-wiring in vulnerable regions, particularly when combined with strategies that reduce hypoxia burden or stabilize the endothelium/NO.

#### 7.6.2. PI3Kγ Signaling in Myeloid Cells as a Reprogramming Lever

Tumor-derived exosomal miR-301a-3p down-regulates PTEN and activates PI3Kγ, reprogramming macrophages toward immunosuppressive states [10]; clinical data with the PI3Kγ inhibitor eganelisib show macrophage reprogramming, immune activation, and ECM remodeling in solid tumors [15]. Given the shared immunometabolic logic between TAMs and plaque macrophages (glycolysis-biased M1, matrix remodeling under hypoxia), selective PI3Kγ inhibition emerges as a plausible strategy to rebalance myeloid programming in atherosclerosis, potentially synergizing with lipid-lowering agents (such as statins) and HIF/NO-targeted approaches [5].

These metabolites and signaling nodes align with the transversal cues discussed across T2D/obesity and cancer.

## 8. Cancer—Hypoxia–HIF–TAM Axis

Cancer remains a leading global health burden: in 2025, an estimated 20.5 million new cancer cases and 9.7 million deaths occurred worldwide, with projections exceeding 30 million annual cases by 2040 [87]. The most prevalent malignancies include breast, lung, colorectal, prostate, and liver cancers, collectively accounting for over 50% of global incidence. Despite therapeutic advances, cancer imposes a significant economic impact, with global costs surpassing USD 1 trillion annually and substantial disparities in survival between high-income and low-income regions.

### 8.1. Tumor Microenvironment (TME)

The tumor microenvironment (TME) comprises immune and stromal cells, vasculature, and the extracellular matrix, which co-evolve with cancer cells to shape growth, invasion, and immune evasion [88,89]. Among immune components, tumor-associated macrophages (TAMs) are abundant and plastic, orchestrating inflammation, angiogenesis, and immune suppression in a context-dependent manner [88,90]. The TME comprises immune and stromal cells, vasculature, and the extracellular matrix, which co-evolve with cancer cells to shape growth, invasion, and immune evasion [88,89,91].

### 8.2. Hypoxia as a Driver of TME Remodeling

Rapid tumor growth outpaces perfusion, creating chronic and cycling hypoxia that stabilizes HIFs, rewires metabolism, and promotes pro-tumorigenic signaling [92,93]. Through HIF-1α/HIF-2α, hypoxia induces angiogenic programs and inflammatory mediators, reshaping cellular composition and macrophage states in poorly vascularized niches [94,95].

### 8.3. TAM Origins and Diversity (Monocytes, TRMs, TEMs)

TAMs originate from two major sources: circulating monocytes and tissue-resident macrophage (TRM) lineages with self-renewal capacity, and their relative contribution varies across tumor types and niches [91,96,97]. Functionally distinct TAM subsets arise from this ontogeny; for example, TIE2-expressing macrophages (TEMs) localize to perivascular niches and promote angiogenesis and vascular perfusion [88,89]. Circulating CD14/CD16 monocyte subsets contribute to the TAM pool but do not map directly onto M1/M2 states, as their polarization is shaped primarily by local metabolic and hypoxic cues within the tumor microenvironment.

TAM lineages and spatial niches in the TME. Circulating monocytes and tissue-resident macrophages (TRMs) populate hypoxic and perivascular niches; TIE2-expressing macrophages (TEMs) support angiogenesis, while TAMs polarize along a functional continuum under microenvironmental cues [88,91,97,98].

### 8.4. Immunometabolism of TAMs (HIF-Programmed)

TAM function is tightly coupled to metabolism: HIF-1α supports glycolysis and inflammatory effector programs, while oxidative metabolism aligns with reparative functions; hypoxia skews macrophage states by stabilizing HIFs and reprogramming glycolytic/mitochondrial flux [99,100]. In specific settings, HIF-1α also sustains immunosuppressive TAM activities, underscoring the context-specific roles [88,101].

#### Functional Consequences of TAM Programs

Under hypoxia, tumor-associated macrophages increase VEGF-A and other pro-angiogenic mediators, while assisting in the breakdown of perivascular extracellular matrix, thereby promoting neovessel formation. High VEGF-A co-localizes with macrophage-rich, poorly perfused tumor regions and associates with an adverse prognosis [102,103,104,105]. Perivascular TAM-derived VEGF-A transiently disrupts endothelial junctions and enables tumor-cell intravasation at tumor microenvironment of metastasis (TMEM) triads, and TMEM density correlates with metastatic recurrence, nominating TAMs as actionable anti-metastatic targets [106,107,108]. TAM-upregulated ARG1 depletes L-arginine and, together with TGF-β–driven fibrosis, establishes a metabolically and mechanically suppressive niche that impairs CD8^+^ T-cell signaling and effector function [109].

### 8.5. Lactate Axis: PKA–CREB–HIF-1α and Immune Checkpoints

Tumor-derived lactate acts as a paracrine signal that drives TAM polarization and VEGF-A induction via PKA/CREB in a HIF-1α-dependent manner [110,111]. Lactate also upregulates PD-L1 on macrophages, thereby enhancing immune evasion and contributing to epigenetic reprogramming (e.g., histone lactylation) that reinforces angiogenesis and suppression [112,113].

### 8.6. Succinate/SUCNR1 Signaling in Tumor-Associated Macrophages (Mechanistic Overview)

Tumor-derived succinate accumulates in hypoxic niches and acts as a dual intracellular and extracellular signal. Intracellular succinate stabilizes HIF-1α by inhibiting PHDs, whereas extracellular succinate activates SUCNR1 on macrophages, engaging Gq-dependent signaling that shapes TAM migration and polarization. Structural and functional studies demonstrate that tumor-derived succinate functions as a potent extracellular cue for macrophages, with SUCNR1 acting as a metabolite-sensing receptor that regulates migratory and pro-angiogenic programs [114,115].

**Figure 6:**
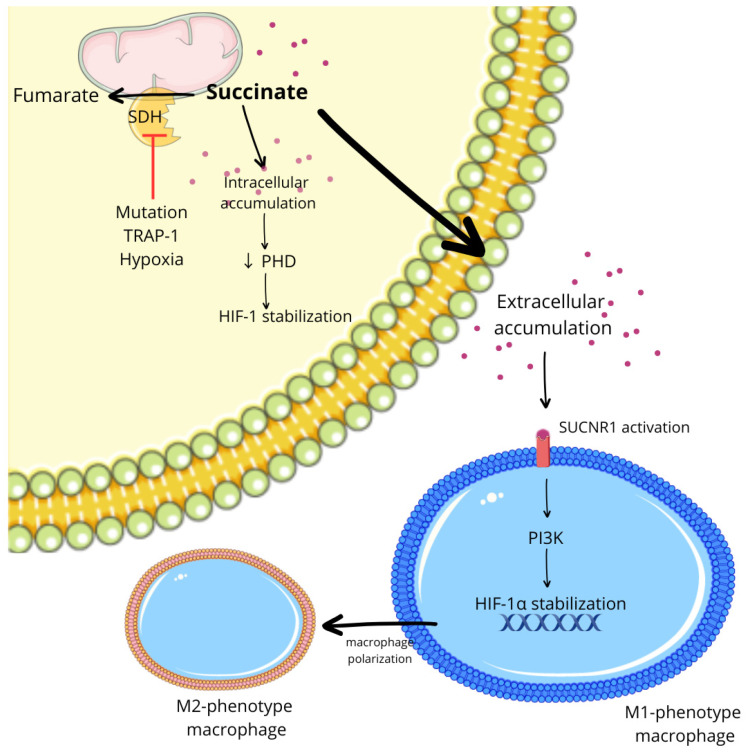
**Succinate-Driven HIF-1α Stabilization and SUCNR1-Mediated Macrophage Polarization.** Succinate accumulation drives HIF-1α stabilization in tumor cells and activates SUCNR1 on macrophages, as reported in [116,117,118]. SDH dysfunction and hypoxia increase intracellular succinate (→ PHD inhibition → HIF-1α stabilization), while extracellular succinate engages SUCNR1 to trigger PI3K/Akt → HIF-1α signaling and M2-like polarization. Parts of the figure were created using images provided by Servier Medical Art (https://smart.servier.com), licensed under CC BY 4. (https://creativecommons.org/licenses/by/4.0/) (Accessed 10 November 2025).

As illustrated in Figure 6, succinate accumulation under hypoxia drives HIF-1α stabilization in tumor cells and activates SUCNR1 signaling in macrophages, thereby reinforcing pro-angiogenic and immunoregulatory programs [116,117,118].

### 8.7. Hypoxia-Regulated Exosomes: PTEN↓ → PI3Kγ↑ and TAM Reprogramming

Hypoxia increases exosome biogenesis and loads oncogenic miRNAs; a canonical example is miR-301a-3p, which suppresses PTEN and activates PI3Kγ, reprogramming macrophages toward immunosuppression and promoting metastasis [119,120]. Related studies indicate that hypoxic tumor exosomes (e.g., miR-21) propagate invasive phenotypes and modulate myeloid cells across various tumor types [121,122]. A comprehensive overview of TAM-mediated mechanisms across solid tumors is provided in several reviews [103,123].

Table 3 summarizes emerging exosome-based therapeutic strategies designed to overcome hypoxia-driven tumor progression and immunosuppression. These approaches exploit exosomes’ natural targeting and biocompatibility to deliver genetic tools (e.g., CRISPR/Cas9), microRNA inhibitors, and chemosensitizers directly to tumor-associated macrophages (TAMs) or hypoxic cancer cells. By modulating key pathways such as PI3Kγ/Akt, HIF-1α signaling, and drug-resistance mechanisms, these strategies aim to reprogram macrophage polarization, restore anti-tumor immunity, and enhance the efficacy of conventional therapies. Recent studies highlight the potential of combining exosome-based delivery with checkpoint inhibitors and anti-HIF drugs to achieve synergistic effects in hypoxic tumor microenvironments [81,91].

### 8.8. Hypoxia → PD-L1 Axis in TAMs (Immune Evasion)

Hypoxia upregulates PD-L1 by activating HIF-1α, which binds to a hypoxia-response element in the PD-L1 promoter, thereby suppressing T-cell activity; this mechanism operates in both tumor cells and TAMs [124]. Clinical studies further link monocyte/macrophage PD-L1 in peritumoral regions with impaired lymphocyte function and worse outcomes [125,126].

HIF-1α binds to a hypoxia-response element in the proximal PD-L1 promoter; in TAMs, this sustains immune evasion and predicts a poor response unless the axis is therapeutically intercepted.

### 8.9. Translational Entry Points (HIF-2α, PI3Kγ, SUCNR1, Exosome-Based Therapies)

Therapeutic strategies targeting the HIF-macrophage axis are emerging as promising interventions across hypoxia-driven pathologies. Selective inhibition of HIF-2α with agents such as belzutifan reduces recruitment and maintenance of M2-like TAMs, thereby mitigating immunosuppressive niches [127]. Similarly, PI3Kγ blockade using eganelisib reprograms macrophages from an M2 to an M1 phenotype and remodels the tumor microenvironment toward immune activation [128]. Antagonism of SUCNR1 interrupts succinate-driven signaling cascades that reinforce M2 polarization under hypoxia, offering another tractable entry point [129]. Exosome-based delivery of anti-miR-301a-3p restores PTEN expression and counters PI3Kγ/Akt signaling in TAMs, reversing immunosuppression and enhancing therapeutic response [10,130]. Finally, PPARγ agonists and SGLT2 inhibitors, though primarily metabolic agents, exert secondary benefits by favoring reparative macrophage programs in hypoxic and inflamed metabolic tissues, linking mechanistic insights from T2D and obesity to oncology [131]. The following subsections outline these translational entry points in greater detail, beginning with HIF-2α inhibitors.

#### 8.9.1. HIF-2α Inhibitors

Belzutifan and related agents demonstrate activity in VHL-driven and sporadic clear-cell renal cell carcinoma (ccRCC), with emerging data supporting their modulation of the immune microenvironment [132,133]. The first-in-class HIF-2α inhibitor PT2385 exhibits target engagement and early clinical signals; preclinical and clinical observations in glioma suggest myeloid remodeling, even in the absence of direct cytotoxicity against tumor cells [134,135,136].

#### 8.9.2. PI3Kγ Inhibition (Myeloid Reprogramming Lever)

PI3Kγ integrates exosome-borne miRNA inputs (for example, miR-301a-3p, which suppresses PTEN), thereby consolidating TAM immunosuppression [137]. Clinical data with eganelisib demonstrate macrophage reprogramming, immune activation, and extracellular matrix remodeling in solid tumors, positioning PI3Kγ as a tractable lever for TAM re-education [138].

#### 8.9.3. Succinate–SUCNR1 Axis (Metabolite–Receptor Node)

From a translational perspective, the succinate–SUCNR1 axis represents a druggable node in hypoxic tumors. Cancer cells accumulate and release succinate, which stabilizes HIF-1α intracellularly and activates SUCNR1 (GPR91) on macrophages, promoting chemotaxis and polarization via Gq-coupled signaling [139,140]. Structural and functional insights support SUCNR1 as a metabolite sensor relevant to migratory and pro-angiogenic myeloid programs [141,142]. This positions SUCNR1 antagonism as a mechanistically coherent approach to remodel TAM states and counter hypoxia-driven immune suppression.

#### 8.9.4. Exosome-Based Therapeutics

In hypoxic tumors, engineered exosome strategies converge on myeloid reprogramming and resistance reversal: anti-miR-301a-3p payloads delivered to TAMs restore PTEN and attenuate PI3Kγ/Akt signaling [10,130,137,143,144]; ligand-decorated exosomes enhance TAM uptake and improve siRNA/drug specificity [145]; combination payloads co-deliver anti-HIF agents with checkpoint inhibitors to counter hypoxia-induced immunosuppression [146]; and CRISPR-loaded exosomes aim at HIF-1α/VEGF or other hypoxia-linked targets to reduce angiogenesis and M2 polarization [147]. A complementary approach utilizes exosome-mediated chemosensitizers to overcome drug resistance under hypoxic conditions [146].

Exosome-based therapeutics—at a glance: anti-miR-301a-3p strategies (aimed at restoring PTEN and attenuating PI3Kγ/Akt signaling in tumor-associated macrophages, thereby reversing immunosuppression [148,149]); TAM-targeting approaches (see comprehensive review [150]), including PI3Kγ inhibitors such as eganelisib, HIF-2α inhibitors such as belzutifan, and SUCNR1 antagonists to block succinate-driven cues [127,129]; co-delivery strategies combining anti-HIF agents with anti-PD-L1/PD-1 to blunt hypoxia-induced checkpoint upregulation and improve response [151]; CRISPR-loaded exosomes designed to edit HIF-1α/VEGF or other resistance nodes in hypoxic tumors [152]; and exosomal chemosensitizers to overcome hypoxia-driven drug resistance and enhance conventional therapy [151]. Future studies should include protocol registration (e.g., PROSPERO) and formal quality assessment as standard practice to improve transparency and reproducibility.

Several emerging strategies complement the core therapeutic approaches discussed above. Engineered macrophages (CAR-M) show promise in early-phase studies, enhancing phagocytosis, remodeling TME, and potentially shifting polarization toward an M1-like state in selected solid tumors [153]. Another avenue involves targeting the C-C chemokine receptor 5 (CCR5) axis: in multiple myeloma models, CCR5 blockade with maraviroc combined with bortezomib reduced M2 polarization and improved chemosensitivity, suggesting a hypothesis-generating approach for TAM-rich settings [154].

Hypoxia imaging also offers translational value. Non-invasive techniques such as ^18F-FMISO PET can localize hypoxic niches that align with HIF-driven macrophage programs in tumors and plaques, supporting risk stratification and therapy monitoring [155]. Additionally, macrophage-expressed dihydropyrimidine dehydrogenase (DPD) can catabolize 5-FU under hypoxic conditions, conferring chemoresistance and nominating the HIF/DPD axis as a potential target [156].

Broad inhibition of HIF pathways (HIF-1α and HIF-2α) and exosome-based anti-miR-301a-3p strategies represent complementary approaches to remodel TAM programs and counter hypoxia-driven immunosuppression.

## 9. Conclusions

Hypoxia stabilizes HIF-1α/HIF-2α and—through lactate, succinate, and hypoxia-regulated exosomes—reprograms macrophages across T2D, atherosclerosis, and cancer, linking oxygen debt to inflammation, tissue remodeling, and immune escape [140,157,158,159]. This macrophage-centered triad explains M1 lock-in in diabetic wounds, glycolysis-biased M1 programs, and cap fragility in atherosclerotic plaques, as well as TAM-mediated angiogenesis and PD-L1-driven suppression in tumors [160,161,162,163]. A concise biomarker panel comprising HIF-1α, VEGF-A, and MMP-9 captures the hypoxia burden and aligns with macrophage state and clinical risk, providing a practical bridge to therapy selection and response monitoring [164,165]. Translational levers—including HIF-2α inhibition, PI3Kγ blockade, SUCNR1 targeting, and exosome-based miRNA modulation—are mechanistically coherent across indications and are well suited for imaging-guided, biomarker-anchored trials [166,167,168,169]. Clinical adoption should focus on reducing the hypoxia burden (endothelium/NO support, wound oxygenation), rebalancing macrophage metabolism (PPARγ/FAO support), and reprogramming myeloid cells in hypoxic tumors to restore antitumor immunity [170,171].

## 10. What Is Novel in This Review

Cross-disease triad. We articulate a single, testable triad—hypoxia → HIF-1α/HIF-2α, lactate/succinate, and hypoxia-regulated exosomes—that coherently explains macrophage reprogramming in T2D/obesity (hypoxic WAT), atherosclerosis (lipid-core hypoxia), and tumors (poorly perfused niches) [6,7,13,24,25].

Macrophage-first integration. We consistently map immunometabolism (glycolysis vs FAO/OXPHOS) onto functional outcomes (M1 lock-in diabetic wounds; plaque vulnerability; TAM-mediated angiogenesis; and immune escape), rather than treating macrophage signals as isolated pathways [4,5,26].

Actionable levers, shared logic. We nominate HIF-2α inhibitors (belzutifan), PI3Kγ inhibitors (eganelisib), SUCNR1 antagonists, and exosomal miRNA modulation as shared levers that can be repurposed across indications, not only in oncology [7,10,14,15].

Compact biomarker panel. We propose a pragmatic biomarker triad—HIF-1α, VEGF-A, and MMP-9—that reflects tissue hypoxia, macrophage phenotype, and downsxplainedtream clinical trajectory. This interpretive pattern is supported by mechanistic, vascular, and neurovascular evidence; we also acknowledge its limitations: circulating HIF-1α is labile, while VEGF-A and MMP-9 lack disease specificity and rise in diverse inflammatory or remodeling contexts [16,20,80].

Translational extensions. Building on this framework, we highlight TAM-expressed DPD as a myeloid driver of 5-FU resistance and [^18F]FMISO PET as a hypoxia-imaging readout aligned with macrophage programming [155,156].

## 11. Clinical and Therapeutic Implications

T2D/obesity and wound care. Strategies that reduce microvascular hypoxia and rebalance macrophage programs—such as SGLT2 inhibitors (which improve endothelial function and nitric oxide bioavailability) and GLP-1 receptor agonists (which reduce inflammation and enhance lipid handling)—can restore the M1-to-M2 transition in chronic diabetic ulcers and promote effective tissue repair [26,57,59,60,62].

Atherosclerosis. The biomarker panel HIF-1α/VEGF-A/MMP-9 aligns with plaque vulnerability and can support the selection and timing of statins and adjunctive therapies, including PPARγ-driven FAO/OXPHOS support and MMP-9 inhibition, to stabilize the fibrous cap [13,16,58].

Oncology. In TAM-rich, hypoxic tumors, combining HIF-2α inhibition or PI3Kγ blockade with immune checkpoint therapy may reprogram TAMs, reduce PD-L1–mediated immune evasion, and sensitize the tumor microenvironment. Exosome-targeted anti-miR-301a-3p represents a precision myeloid-editing strategy for miRNA-high, hypoxia-driven cancers [10,14,15,124].

Imaging-guided care. [^18F]FMISO PET localizes hypoxic niches in plaques and tumors and can be integrated with the HIF-1α/VEGF-A/MMP-9 panel to personalize therapy intensity and monitor macrophage reprogramming over time [86].

## 12. Future Research

Prospective validation. Multi-cohort studies should validate the HIF-1α/VEGF-A/MMP-9 panel across T2D-related wounds, carotid and coronary atherosclerosis, and solid tumors, linking longitudinal measurements to macrophage phenotyping and clinical endpoints [16,80].

Interventional trials. PI3Kγ inhibitors (macrophage reprogramming) and SUCNR1 modulators (succinate signaling) warrant testing in plaque biology and selected solid tumors, ideally with macrophage-state readouts and hypoxia-imaging endpoints [7,8,15].

Exosome/miRNA precision strategies. Phase I/II studies should evaluate anti-miR-301a-3p delivery to TAMs, including pharmacodynamic effects on PTEN/PI3Kγ and PD-L1, as well as targeting performance and safety [10,11].

Wound-repair mechanistic trials. Oxygenation devices combined with SGLT2 inhibitors or GLP-1 receptor agonists merit investigation, with attention to temporal HIF switching and quantitative M1-to-M2 metrics [26,57].

Assay standardization. Harmonization of HIF-1α/HIF-2α, VEGF-A, and MMP-9 assay platforms is needed, along with the definition of disease-specific clinical cut points and their imaging correlates [16,86].

## 13. Methodological Considerations and Limitations

Macrophage states exist along a dynamic continuum rather than within a strict M1/M2 dichotomy, and their functional outputs vary with disease stage, tissue oxygenation, and therapeutic context [4,5]. This complexity requires cautious extrapolation across experimental models and disease settings.

Translational challenges also remain substantial. Approaches such as SUCNR1 inhibition or exosome-based interventions require further progress in druggability and biomanufacturing maturity, and potential off-target effects or immunogenicity mandate rigorous monitoring [8,11]. These issues highlight the gap between mechanistic insights and clinical implementation.

In addition, the current evidence base is heterogeneous, with many findings derived from preclinical models. Ensuring clinical relevance will require validation in human studies using standardized biomarkers and hypoxia-imaging endpoints [15,86], which is essential for bridging experimental observations with patient-centered applications.

Finally, although the search strategy was comprehensive, this review did not involve protocol registration (e.g., PROSPERO) and did not include a formal assessment of study quality or risk of bias. These omissions increase the potential for selection bias and limit reproducibility. Furthermore, the narrative design precludes quantitative synthesis, and heterogeneity across models and assays constrains generalizability. Future work should incorporate standardized quality-appraisal tools and protocol registration to strengthen methodological rigor.

## Figures and Tables

**Table 1:** Hypoxia-induced pathways and macrophage polarization—concise cross-condition map.

Condition	Key Hypoxic Pathways and Regulators	Molecular and Metabolic Effects	Macrophage Polarization and Impact	References
Type 2 diabetes	HIF-1α stabilization; decreased HIF-1β (ARNT) in islets; SGLT2-related modulation	Reduced ATP synthesis and impaired GSIS; increased pro-inflammatory cytokines	Predominant M1 profile; in diabetic wounds, failure of the normal transition from M1-to-M2, leading to a chronic non-healing trajectory	[1,24,25,26,27]
Obesity	WAT hypoxia; CCR2/MIF signaling; MCP-1/MIP-1α- mediated monocyte recruitment	Increased Leptin, IL-6 and TNF-α; decreased adiponectin; insufficient vascularization	Enhanced monocyte infiltration with differentiation into M1-like ATMs, sustaining systemic inflammation and insulin resistance	[12,24]
Atherosclerosis	HIF-1α stabilization in lipid core; NF-κB/ROS activation; reduced ABCA1	Increased glycolysis (GLUT1/HK/PFK); reduced cholesterol efflux	M1 macrophages accumulate at plaque shoulders; MMP-2/9-driven ECM degradation weakens the fibrous cap and increases plaque vulnerability	[5,13,19]
Cancer	HIF-1α/HIF-2α activation; lactate-PKA–CREB signaling; Succinate-SUCNR1 signaling; hypoxic exosomal miR-301a-3p	Warburg metabolic shift; increased VEGF, ARG1 and IL-10; ECM digestion via MMP-9, cathepsins	TAMs polarize toward M2/M2d-like immunoregulatory states (angiogenic VEGF-high), promoting angiogenesis, immune evasion, and metastatic progression.	[6,7,8,9,10,11]

*Abbreviations:* ABCA1—ATP-binding cassette transporter A1; ARG1—arginase-1; ARNT—aryl hydrocarbon receptor nuclear translocator (HIF-1β); ATP—adenosine triphosphate; CREB—cAMP response element-binding protein; ECM—extracellular matrix; GLUT1—glucose transporter 1; GSIS—glucose-stimulated insulin secretion; HIF-1α—hypoxia-inducible factor 1 alpha; HIF-1β—hypoxia-inducible factor 1 beta; HIF-2α—hypoxia-inducible factor 2 alpha; HK—hexokinase; IL-6—interleukin-6; IL-10—interleukin-10; M2d—angiogenic/immunoregulatory TAM-like subtype of macrophages; MCP-1—monocyte chemoattractant protein-1; MIF—macrophage migration inhibitory factor; MIP-1α—macrophage inflammatory protein-1 alpha; MMP-2—matrix metalloproteinase-2; MMP-9—matrix metalloproteinase-9; NF-κB—nuclear factor kappa-light-chain-enhancer of activated B cells; PFK—phosphofructokinase-1; PKA—protein kinase A; ROS—reactive oxygen species; SGLT2—sodium–glucose cotransporter-2; SUCNR1—succinate receptor 1; TAM—tumor-associated macrophage; TNF-α—tumor necrosis factor alpha; VEGF—vascular endothelial growth factor.

**Table 3:** Emerging Exosome-Based Therapeutic Strategies Targeting Hypoxia-Driven Tumor Progression and TAM Reprogramming.

Strategy	Target	Mechanism	Potential Clinical Application	References
Anti-miR-301a-3p delivery via engineered exosomes	PTEN/PI3Kγ axis	Restores PTEN, attenuates PI3Kγ/Akt signaling	Reprogram TAMs; reduce immunosuppression	[119]
Ligand-decorated exosomes for TAM targeting	Surface receptors on TAMs: CD206 (MRC1), CD163, TIE2, PD-L1 (CD274), SUCNR1 (GPR91)	Enhances uptake and specificity	Improves siRNA/drug delivery	[120]
Combination payloads (anti-HIF + checkpoint inhibitors)	HIF-1α, PD-L1	Blocks hypoxia- induced immune escape	Synergizes with immunotherapy	[121]
CRISPR-loaded exosomes	HIF-1α/VEGF genes	Gene editing to reduce angiogenesis	Counters hypoxia-driven resistance	[122]
Exosomal chemosensitizers	Drug-resistance pathways	Overcomes hypoxia-induced chemoresistance	Enhances conventional therapy	[123]

*Abbreviations:* Akt—protein kinase B; CD163—cluster of differentiation 163; CD206 (MRC1)—mannose receptor C-type 1; CRISPR—clustered regularly interspaced short palindromic repeats; GPR91 (SUCNR1)—succinate receptor 1; HIF-1α—hypoxia-inducible factor 1 alpha; miR—microRNA; PD-L1 (CD274)—programmed death-ligand 1; PI3Kγ—phosphoinositide 3-kinase gamma; PTEN—phosphatase and tensin homolog; siRNA—small interfering RNA; TAM—tumor-associated macrophage; TIE2—tyrosine kinase with immunoglobulin-like and EGF-like domains 2; VEGF—vascular endothelial growth factor.

## Data Availability

Not applicable. No new data were created or analyzed in this narrative review.

## Abbreviations

ABCA1	ATP-binding cassette transporter A1
AGEs	advanced glycation end-products
ARG1	arginase-1 (M2 macrophage marker; arginine catabolism)
ARNT	aryl hydrocarbon receptor nuclear translocator (HIF-1β)
ATP	adenosine triphosphate
cAMP	cyclic adenosine monophosphate
CAR-M	chimeric antigen receptor macrophages
CCL2	C-C motif chemokine ligand 2 (MCP-1)
CCL3	C-C motif chemokine ligand 3 (MIP-1α)
CCR2	C-C chemokine receptor 2
CCR5	C-C chemokine receptor 5
ccRCC	clear-cell renal cell carcinoma
CD163	cluster of differentiation 163
CD206	cluster of differentiation 206
CD274	programmed death-ligand 1 (PD-L1)
CPT1	carnitine palmitoyltransferase 1
CREB	cAMP response element-binding protein
CRISPR	clustered regularly interspaced short palindromic repeats
DAMPs	damage-associated molecular patterns
DPD	dihydropyrimidine dehydrogenase
ECM	extracellular matrix
EMT	epithelial–mesenchymal transition
FAO	fatty acid oxidation
FMD	flow-mediated dilation
FMISO	[18F]-fluoromisonidazole
FMISO PET	fluoromisonidazole positron emission tomography
GLUT1	glucose transporter 1
GPR91	succinate receptor 1 (SUCNR1)
GSIS	glucose-stimulated insulin secretion
HIF	hypoxia-inducible factor
HIF-1	hypoxia-inducible factor 1
HIF-1α	hypoxia-inducible factor 1 alpha
HIF-1β	hypoxia-inducible factor 1 beta (ARNT)
HIF-2	hypoxia-inducible factor 2
HIF-2α	hypoxia-inducible factor 2 alpha
HIF-α	HIF alpha subunit
HK	hexokinase
IMT	intima–media thickness
IL-1β	interleukin-1 beta
IL-4	interleukin-4
IL-6	interleukin-6
IL-10	interleukin-10
IL-13	interleukin-13
LDL	low-density lipoprotein
M1	classically activated macrophages
M2	alternatively activated macrophages
M2d	angiogenic/immunoregulatory subtype of macrophages
MCP-1	monocyte chemoattractant protein-1
MDSC	myeloid-derived suppressor cell(s)
MIF	macrophage migration inhibitory factor
MIP-1α	macrophage inflammatory protein-1 alpha
miR	microRNA
MMP	matrix metalloproteinases
MMP-2	matrix metalloproteinase 2
MMP-9	matrix metalloproteinase 9
NADPH	nicotinamide adenine dinucleotide phosphate (reduced)
NF-κB	nuclear factor kappa-light-chain-enhancer of activated B cells
NO	nitric oxide
NOX4	NADPH oxidase 4
OGTT	oral glucose tolerance test
OXPHOS	oxidative phosphorylation
PD-1	programmed cell death protein 1
PD-L1	programmed death-ligand 1
PDH	pyruvate dehydrogenase
PET	positron emission tomography
PFK	phosphofructokinase-1
PFKFB3	6-phosphofructo-2-kinase/fructose-2,6-bisphosphatase 3
PHD	prolyl hydroxylase domain
PHD2	prolyl hydroxylase domain 2
PI3K	phosphoinositide 3-kinase
PI3K/AKT	PI3K–Akt signaling pathway
PI3Kγ	phosphoinositide 3-kinase gamma
PKA	protein kinase A
PKA/CREB	PKA–CREB pathway
PKC	protein kinase C
PPARγ	peroxisome proliferator-activated receptor gamma
PT2385	HIF-2α inhibitor (PT2385)
PTEN	phosphatase and tensin homolog
PTEN/PI3Kγ	PTEN–PI3Kγ axis
RAs	receptor agonists (GLP-1 RAs)
ROS	reactive oxygen species
SDH	succinate dehydrogenase
SGLT	sodium–glucose cotransporter family
SGLT2	sodium–glucose cotransporter 2
siRNA	small interfering RNA
STAT6	signal transducer and activator of transcription 6
SUCNR1	succinate receptor 1
T2D	type 2 diabetes
TAM	tumor-associated macrophage
TCA	tricarboxylic acid
TG	triglycerides
TGF-β	transforming growth factor beta
TIMP	tissue inhibitor of metalloproteinases
TLR	Toll-like receptor
TLR4	Toll-like receptor 4
TME	tumor microenvironment
TMEM	tumor microenvironment of metastasis
TNF	tumor necrosis factor
VEGF	vascular endothelial growth factor
VEGF-A	vascular endothelial growth factor A
VHL	von Hippel–Lindau protein
WAT	white adipose tissue
